# Revisiting ameloblastin; addressing the EMT-ECM axis above and beyond oral biology

**DOI:** 10.3389/fcell.2023.1251540

**Published:** 2023-11-13

**Authors:** Janne E. Reseland, Catherine A. Heyward, Athina Samara

**Affiliations:** ^1^ Center for Functional Tissue Reconstruction (FUTURE), University of Oslo, Oslo, Norway; ^2^ Department of Biomaterials and Oral Research Laboratory, Faculty of Dentistry, University of Oslo, Oslo, Norway

**Keywords:** ameloblastin, extracellular matrix, epithelial–mesenchymal transition, endothelial–mesenchymal transition–extracellular matrix axis, gastrointestinal tract, tonsils, esophagus, stomach

## Abstract

Ameloblastin (AMBN) is best characterized for its role in dental enamel formation, regulating cell differentiation and mineralization, and cell matrix adhesion. However, AMBN has also been detected in mesenchymal stem cells in addition to bone, blood, and adipose tissue. Using immunofluorescence in a pilot scheme, we identified that AMBN is expressed in different parts of the gastrointestinal (GI) tract. AMBN mRNA and protein detection in several tissues along the length of the GI tract suggests a role for AMBN in the structure and tissue integrity of the extracellular matrix (ECM). Intracellular AMBN expression in subsets of cells indicates a potential alternative role in signaling processes. Of note, our previous functional AMBN promoter analyses had shown that it contains epithelial–mesenchymal transition (EMT) regulatory elements. ΑΜΒΝ is herein presented as a paradigm shift of the possible associations and the spatiotemporal regulation of the ECM regulating the EMT and *vice versa*, using the example of AMBN expression beyond oral biology.

## Introduction

## AMBN beyond oral biology

Ameloblastin (AMBN) is a pleiotropic protein (Spoutil et al., 2023) previously assumed to be specifically expressed by ameloblasts and responsible for extracellular crystal formation of enamel during tooth development (Lacruz et al., 2017; Liang et al., 2019). While AMBN-related research has primarily focused on dental tissues, potential AMBN functions in non-oral tissues and organs have been suggested (Tamburstuen et al., 2011; Stakkestad et al., 2018; Kegulian et al., 2023). During embryonic development, it can be present in salivary glands (Sharifi et al., 2021) and also in adult adipose tissue (Stakkestad et al., 2018). Detection in the bone-forming cells (osteoblasts), influencing osteoblast activity and mineralization (Tamburstuen et al., 2011; Lu et al., 2016; Stakkestad et al., 2017; Spoutil et al., 2023), indicates the role of AMBN in bone development and repair.

Characterization of AMBN-KO mouse models demonstrated that skeletal–muscular function and red blood cell differentiation were affected (Liang et al., 2019) and identified differential expression of AMBN in cardiac cells during embryonic heart development (Masino et al., 2004; Masino et al., 2005). Furthermore, AMBN was detected by transcriptomics and *in situ* mRNA hybridization in a subpopulation of basal cells (Andrew et al., 2023) on zebrafish skin, and the cells previously mentioned have a secretory morphology in contact with the calcified scale matrix (Sire et al., 1997). Information provided by commercial producers (Santa Cruz Inc, 2023) of antibodies against AMBN also refers to immunodetection in various tissues, such as cytoplasmic staining of cells in glomeruli. However, the precise AMBN function in these organs or in the tissue development and physiology is not yet fully understood.

## Is there a link between tissue structure and AMBN expression?

The intestinal ECM acts as a barrier to the external environment, providing protection and mechanical support to cells and elasticity and resistance to tensile forces on organs (Pompili et al., 2021). The ECM provides structural support and integrity to the cells lining the GI tract and as a scaffold holds the cells together and maintains the tissue structural organization, and it provides an insight to cell–ECM interactions (Onfroy-Roy et al., 2020). These signaling and physical cues influence cell behavior, including adhesion, migration, and differentiation, and ECM-centered studies can reveal how it regulates various cellular processes and contributes to tissue homeostasis and repair. Tissue-specific ECM rearrangements create structures and spaces for unique cellular processes, such as intestinal villi and crypts containing stem cell regeneration pools (Yue, 2014; Pennings et al., 2018). Numerous factors regulating ECM composition and structure are known, but do we know them all and how tissue-specific they are? Understanding the composition and function of the ECM in the GI tract helps elucidate the mechanisms that contribute to tissue stability and integrity.

**FIGURE 1:**
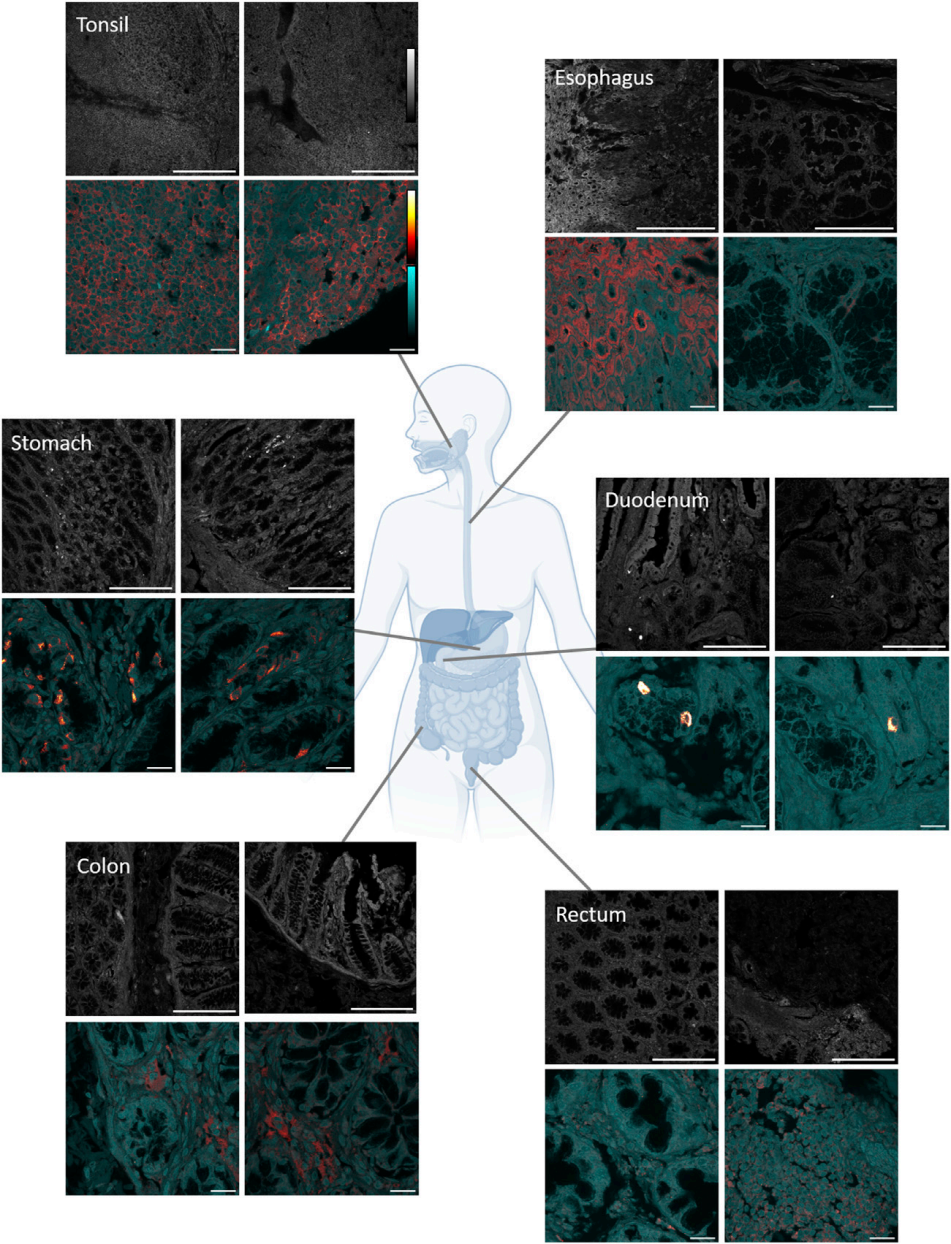
Immunofluorescence imaging of various GI tract tissues. Upper grayscale panels show overview of the tissue in the region where the AMBN signal was detected. Scale bar 200 μm. Lower panels show detection of AMBN immunostaining shown in ImageJ lookup table “red hot” and tissue autofluorescence in cyan. Scale bar 20 μm. GI tract overview diagram was created in Biorender.

AMBN expression in soft tissues of the gastrointestinal tract was examined by indirect immunofluorescence on a commercial human tissue array of normal adult formalin-fixed paraffin-embedded tissues. Tissue cores were imaged first to give an overview of the tissue sample and then were imaged at a higher magnification to focus on the AMBN distribution. AMBN was detected in the tonsil, esophagus, stomach, duodenum, colon, and rectum (Figure 1), although its expression levels and localization varied (negative controls without the primary antibody are shown in the Supplementary Figure S1.).

In the tonsil, many cells expressed a low level of AMBN. There was no particular localization or grouping of the AMBN-positive cells across the tonsil tissue core (diameter 1.5 mm) and little variation in AMBN expression. In contrast, in the esophagus, AMBN was predominantly expressed in squamous epithelium. Here, there were clear demarcations between the AMBN-positive cells in the squamous epithelium and the AMBN-negative cells in the submucosa, although the expression level within the squamous epithelium varied. Epithelium AMBN expression was confirmed in rat gingiva, using a different anti-AMBN antibody (Supplementary Figure S2).

In the stomach, the expression of AMBN was restricted to groups of cells within the mucosa, suggesting it may mark specialized cell subgroups. These groups were among the gastric pits, although there was no obvious pattern for the number and distribution of the AMBN-positive cells. In the duodenum, few cells expressed detectable AMBN, but at a much higher level than that seen in the other GI tissues (demonstrated by the saturation of the high-magnification image set of the duodenum in Figure 1, due to the high level of AMBN expression relative to the other tissues, at constant imaging conditions). The stomach and duodenum cells had more punctate AMBN distribution within the cells, suggesting a vesicular intracellular localization. In the colon, the AMBN expression was again more diffuse, localizing mainly between mucosal crypts. In the rectum, low levels of AMBN were present in both the mucosa and submucosa, with no particular AMBN expression pattern. Both these tissues demonstrated low levels of AMBN, where the protein may be cytosolic and/or part of the extracellular matrix.

**FIGURE 2:**
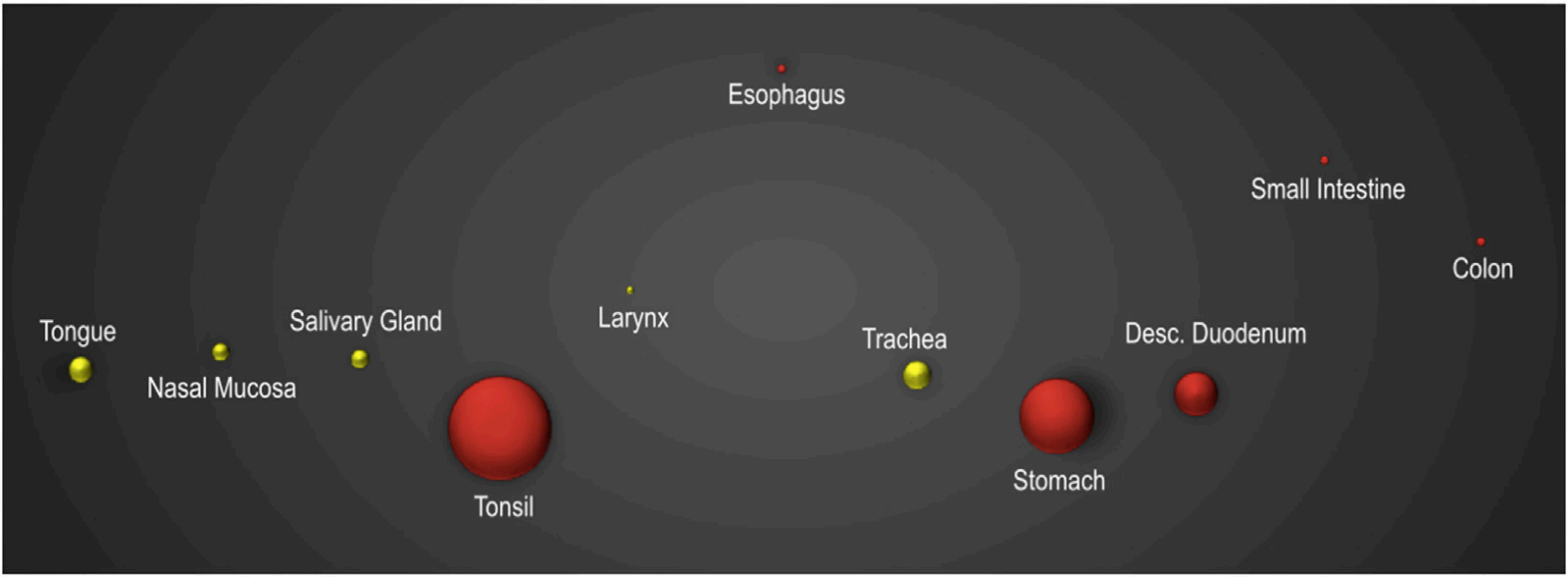
qRT-PCR analysis of various oral and GI tract tissues: a commercial cDNA array containing human tissue samples from the oral and gastrointestinal (GI) tract tissue was employed as the basis for a quantitative real-time polymerase chain reaction (qRT-PCR) analysis to assess the mRNA levels of *AMBN* across a spectrum of tissues within these anatomical regions. In the graphical representation of the data, bubbles indicate the calculated 2^-(∆∆C_T_) values, normalized to the expression of the housekeeping gene GAPDH. The PCR confirmed AMBN expression in tonsils, esophagus, stomach, and intestine (in red) and further showed that it is abundantly expressed in the human tongue, nasal mucosa, salivary glands, larynx, and trachea (as shown in the yellow bubbles).

To complement and confirm the protein data, a commercially available human tissue cDNA array was used to determine the expression profile of AMBN mRNA, in tissues of the oral cavity and along the GI tract (Figure 2). The PCR confirmed *AMBN* expression in the tonsils, esophagus, stomach, and intestine (in red) and further showed that it is abundantly expressed in the human tongue, nasal mucosa, salivary glands, larynx, and trachea (as shown in the yellow bubbles).

## AMBN in the GI tract: a paradigm shift?

In this pilot study, we aimed to map the protein expression of AMBN in healthy adult tissue beyond the oral cavity. Using immunofluorescence, we identified the AMBN expression in the GI tract and documented its presence in the tonsils, esophagus, stomach, and intestines. The expression pattern demonstrated in the tonsil, esophagus, colon, and rectum is consistent, suggesting a role for AMBN as a structural component of the ECM, particularly in regions subjected to mechanical stress, such as shear forces and stretching (Janssen et al., 2011). The more dispersed nature of the AMBN-positive cells in the stomach and duodenum and contrasting intracellular localization could however suggest alternative proteolytic products and/or alternative function(s) (Iwata et al., 2007; Vetyskova et al., 2020).

*AMBN* expression has been previously documented (Tamburstuen et al., 2011) to be high in CD34^+^ mesenchymal stem cells (MSCs), which are a major component of the intestinal stem cells niche (Stzepourginski et al., 2017). Of note, RNA-seq analysis has documented the AMBN expression in the human intestine (Protein Atlas, 2023) but not in a murine model of intestinal epithelium organoids (Ohsaka and Sonoyama, 2018). Thus, the high protein expression of AMBN within specific cell subpopulation of cells in the stomach and duodenum could suggest specific spatiotemporal roles and alternative functions related to the gastric stem cell niche. We thus suggest that charting AMBN as an integral component of the ECM is crucial to understand the role of the extracellular matrix (ECM) in specific cell clusters and GI tract lining. This may be crucial for the deconvolution of the partners of tissue structure and integrity.

## AMBN and the epithelial–mesenchymal transition (EMT)

**FIGURE 3:**
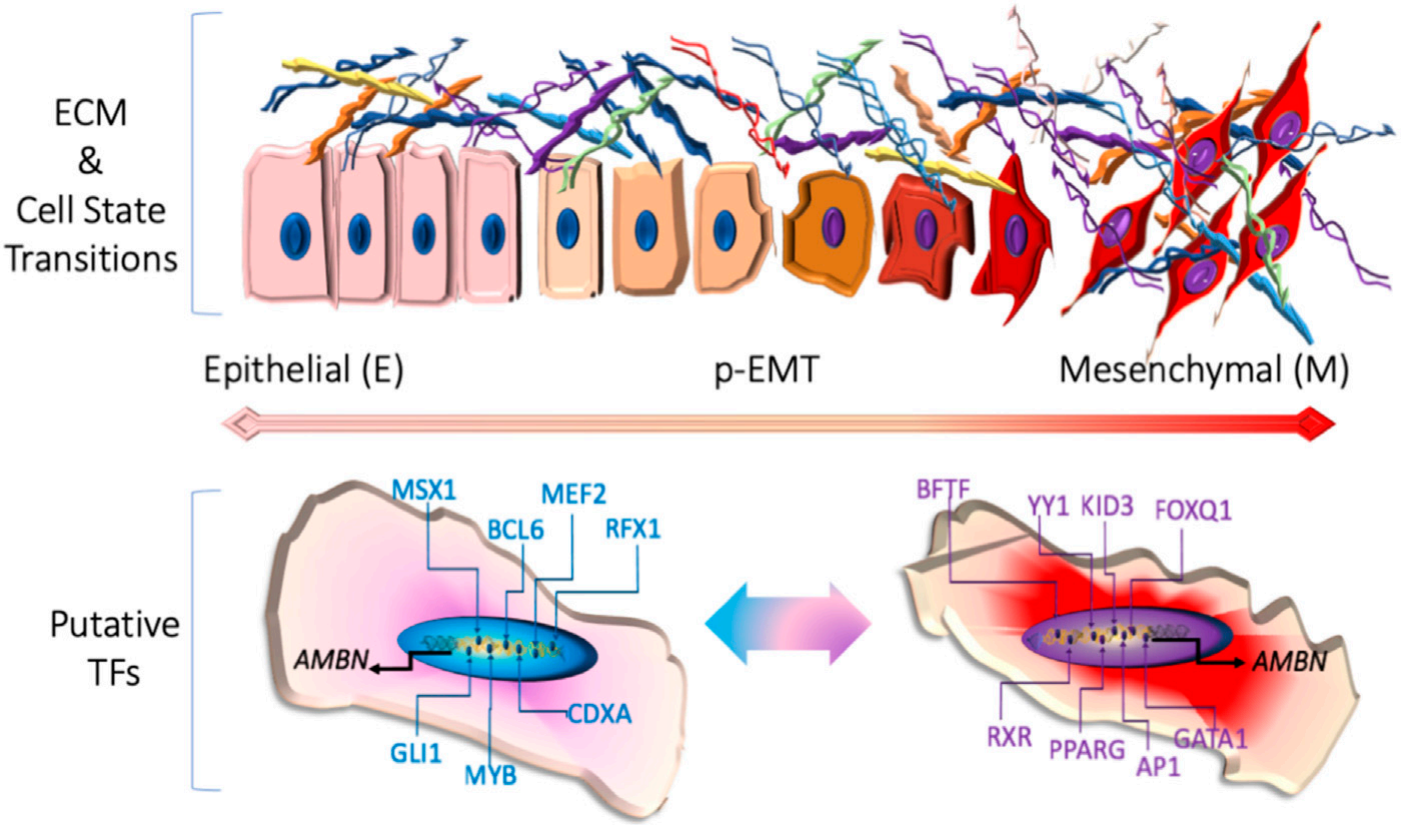
Upper panel: cellular changes during epithelial-to-mesenchymal transition (EMT). Lower panel: putative transcription factors (TFs) and/or promoter elements related to GI tract (left) and EMT (in purple font) AMBN transcription regulation.

AMBN has previously been linked to critical signaling roles in the initiation of cell polarity (Visakan et al., 2022) and in cell-to-cell and cell-to-matrix interactions (Su et al., 2020). The signals from the ECM are known to be key regulators of epithelial–mesenchymal transition (EMT) both in physiology and disease (Scott et al., 2019) and may vary from gene expression modifications to epigenetic reprogramming cues (Peixoto et al., 2019). EMT is a tiered process where epithelial cells partially lose their epithelial characteristics, such as polarity and adhesiveness, and acquire MSC properties (Kalluri and Weinberg, 2009; Xiang et al., 2022). It is key among the processes taking place in cells of ectodermal and epithelial origins that shape the tooth structures and contribute to the cells involved in developing teeth, connect them to the bone, and help them remain embedded in the jaw (Hermans et al., 2021). EMT is suggested to be a sequential process, where the cell goes through an intermediate state, termed partial (p)-EMT; at that state, the cells have both E and M traits (hybrid E/M) and high degree of plasticity (Kalluri and Weinberg, 2009; Aiello et al., 2018) (Figure 3).

Of note, we know that of the chain of reciprocal epithelial–mesenchymal interactions regulating tooth morphogenesis and tooth-specific cell differentiation, including odontoblasts, ameloblasts, and cementoblasts, the role of AMBN in EMT and pEMT is not delimited.

## AMBN promoter: old insights and new routes

We had previously investigated the AMBN promoter and identified regulatory elements associated with the EMT (Tamburstuen et al., 2011) (Figure 3). Among them were potential binding sites for transcription factors associated with intestinal MSC regulation, such as MYB (Cheasley et al., 2011; Cicirò and Sala, 2021), identified in addition to the intestine ectopic crypt and cell differentiation markers MSX1 (Horazna et al., 2019) and CDX1. The latter is also expressed in intestinal epithelium MSCs and in the crypts and base of villi of the differentiating cells replacing the senescent cells with digestive activity (Frumkin et al., 1994). Strikingly, among the putative promoter elements, we mapped transcription factors associated with EMT in physiology and oncogenesis, such as RFX1 (Isaac et al., 2021), BCL6 (Yang et al., 2020), GLI1 (Lei et al., 2022), and OCT1 (Li et al., 2015). Another AMBN promoter analysis (Smith et al., 2020) identified SP6, later suggested to be among the genes implicated in gastric cancer (Zhou et al., 2021) together with MEF2, which resulted from our own AMBN promoter analysis. Our own recent data have associated AMBN expression levels with testicular (Geng et al., 2022) and ovarian cancer (Geng et al., 2023). Thus, the ECM–EMT AMBN association and the GI tract localization data shed a different light on the old promoter data, and the role of these regulatory elements shall be unveiled in future studies.

## New AMBN perspectives

It is known that during mineralization of enamel, AMBN is processed into smaller fragments, adding to the puzzle of the spatiotemporal and distinct roles of AMBN isoforms (Ravindranath et al., 2007; Wazen et al., 2009; Smith et al., 2009; Stakkestad et al., 2018). Thus, the different localization patterns we see in the GI tract may also reflect different proteolytic products from the full-length AMBN protein, which are still recognized by the same antibody. As the distribution and expression level of AMBN differed greatly between the tissues, this could again suggest that AMBN may play more than one role in the GI tract. Understanding the role of AMBN in the ECM of the cells lining the GI tract is crucial for comprehending tissue structure, cell behavior, barrier function, and disease pathogenesis.

The EMT is associated both with the development of normal intestinal tissues and human inflammatory bowel disease (Brabletz et al., 2009; Flier et al., 2010; Jiang et al., 2018; Lovisa et al., 2019). Of note, the epithelial cells lining the GI tract are responsible for the formation of the protective barrier separating the internal environment from the external environment, responsible for food passage (Okumura and Takeda, 2017). The role of the ECM is instrumental in maintaining the integrity of this barrier. Thus, to identify and understand the interactions of AMBN as an ECM component of lining cells in physiology would shed light into the mechanisms of barrier function.

## Concluding remarks

Lamppost findings are often used to map step-by-step research routes, indicating prior discoveries and guided by cost-effectiveness. However, in the context of the gastrointestinal (GI) tract ECM, a noteworthy paradigm shift emerges with the introduction of AMBN, beyond the traditional routes. This protein, conventionally recognized as a tooth ECM-associated partner, warrants a fresh perspective.

In this age of extensive data and innovative single-cell methodologies, we have the opportunity to explore uncharted pathways. The preliminary results discussed here provide insights into the structural components governing GI tract physiology. Furthermore, they showcase possible associations of the spatiotemporal regulation of the ECM regulating the EMT and *vice versa*, using the example of AMBN beyond oral biology. We present AMBN as an example of how the body spatiotemporally recycles genes to uphold tissue reconstruction and homeostasis. Undoubtedly, future studies on the expression and role of AMBN in pathophysiological phenomena could lead to advancements in diagnostics, therapeutics, and regenerative medicine.

Herein, we aimed to broaden the EMT field with a systems biology perspective and suggest that the EMT–ECM axis needs to be addressed as a composite entity in both developmental biology and regenerative medicine.

## Data Availability

The original contributions presented in the study are included in the article/Supplementary Material; further inquiries can be directed to the corresponding author.

## Supplementary material

SUPPLEMENTARY FIGURE S1 Negative control images for immunofluorescence labeling of normal human adult tissue array. Negative control samples were treated as for test samples, except that the primary antibody was omitted. Scale bar 20 μm. Autofluorescence and immunolabelling images are both shown in grayscale.

SUPPLEMENTARY FIGURE S2 Rat gingiva formalin-fixed paraffin-embedded sections stained by indirect autofluorescence with anti-AMBN (DF8503, 1:200, Affinity Biosciences) and goat anti-rabbit Alexa Fluor 488 **(A–C)** or negative control where the primary antibody was omitted **(D–F)**. Panels show tissue autofluorescence **(A, D)**, Alexa Fluor 488 signal **(B, E)**, or merged images **(C, F)**. Merged images show autofluorescence in cyan and Alexa Fluor 488 signal in ImageJ lookup table “red hot”. Scale bar 20 μm.
