# Supplementary figures and images for: Revisiting ameloblastin; addressing the EMT-ECM axis above and beyond oral biology
Source: Front Cell Dev Biol. 2023 Nov 13;11:1251540. doi: 10.3389/fcell.2023.1251540 (PMC10679718; doi:10.3389/fcell.2023.1251540)

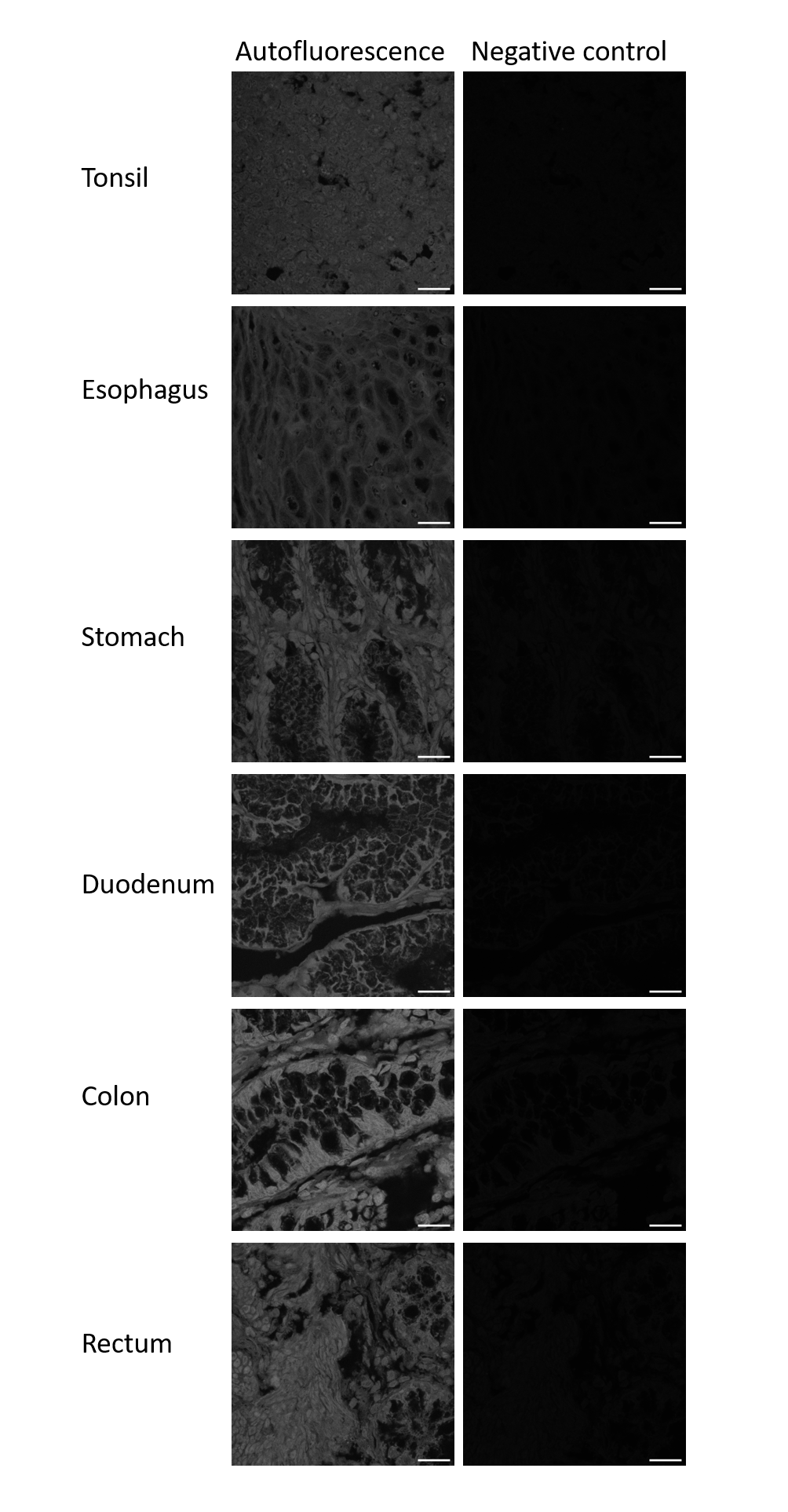

Supplement: Supplementary file 1 [file Image-1.tiff]

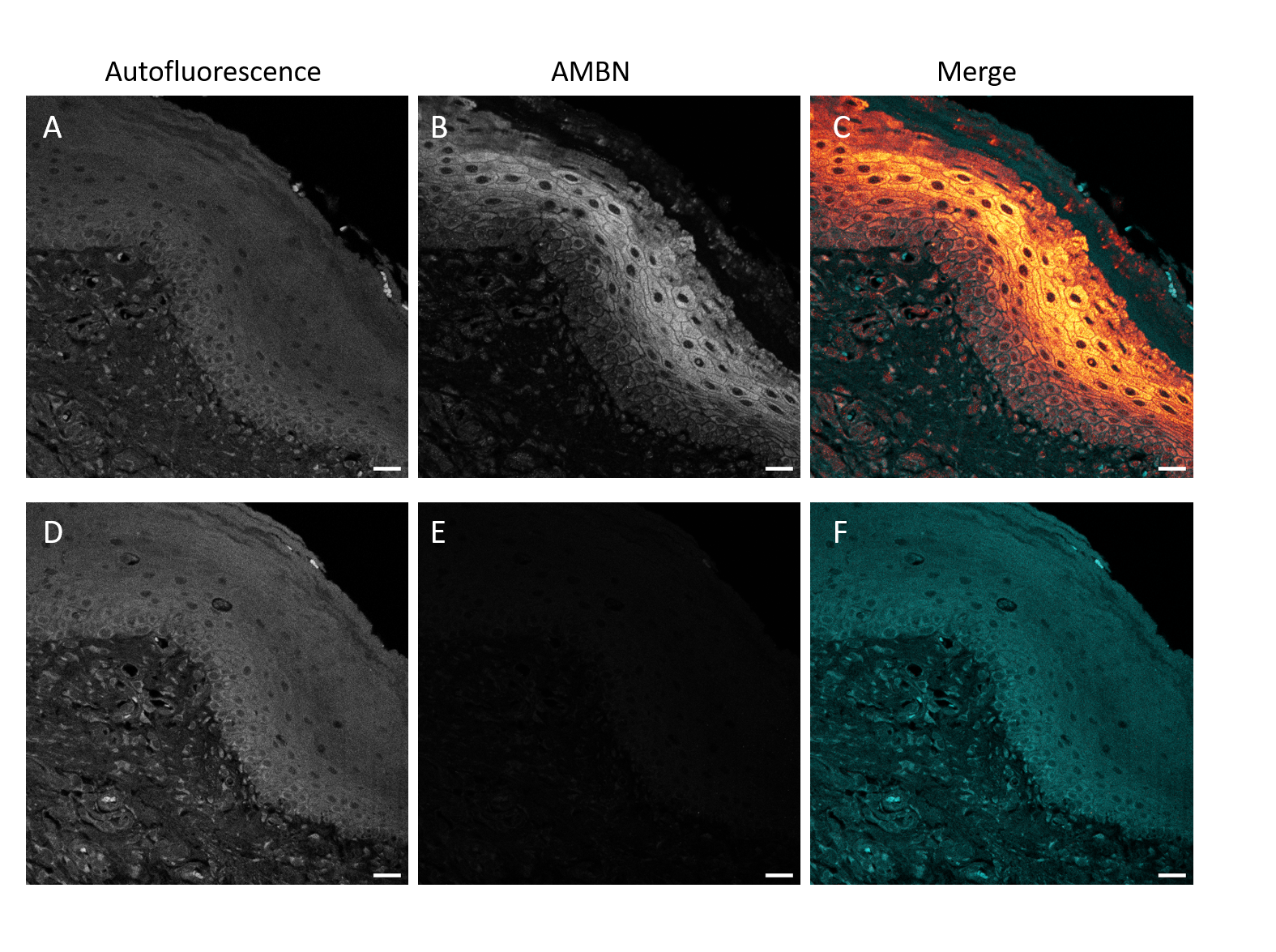

Supplement: Supplementary file 2 [file Image2.tiff]
