# Supplementary material for: Revisiting ameloblastin; addressing the EMT-ECM axis above and beyond oral biology
Source: Front Cell Dev Biol. 2023 Nov 13;11:1251540. doi: 10.3389/fcell.2023.1251540 (PMC10679718; doi:10.3389/fcell.2023.1251540)
Supplement: Supplementary file 3 [file DataSheet1.docx]

***Revisiting ameloblastin; addressing the EMT-ECM--axis beyond and above oral biology***

Janne E Reseland^1,2^, Catherine A Heyward^2^, and Athina Samara^1.2,^

^1^Center for functional tissue reconstruction (FUTURE);

^2^Department of Biomaterials and Oral Research Laboratory, Faculty of Dentistry, University of Oslo, Oslo, Norway

**Correspondence:**Athina Samara, [athinas@uio.no](mailto:athinas@uio.no)

**Supplementary Data**

**Materials and Methods in brief**

We used a human adult normal formalin-fixed paraffin-embedded (FFPE) tissue array slides (T8234708-2) were purchased from AMSBio (Abingdon, UK). Anti-AMBN sc-271012 (H-2) raised in mouse was from Santa Cruz (Dallas, Texas, USA). Anti-AMBN DF8503 was purchased from Affinity Biosciences. Alexa Fluor 488-conjugated goat anti-mouse secondary antibody (A11034) was from Thermo Fisher Scientific (Waltham, Massachusetts, USA). Normal goat serum (NGS) (ab7481) was from Abcam (Cambridge, UK). All other reagents were purchased from Sigma Aldrich (St. Louis, Missouri, USA).

**Immunolabeling**

Human tissue array slides were baked at 60°C for 30 minutes before deparaffinizing in xylene and rehydrating through a graded ethanol series to water. Heat mediated antigen retrieval was carried out for 20 minutes at 95°C in 10mM citrate buffer pH6, 0.05% Tween 20. Slides were allowed to cool to room temperature before rinsing in phosphate buffered saline (PBS) and treating with 0.1% Triton X-100 in PBS for 5 minutes. Samples were washed in PBS three times then blocked in 10% normal goat serum (NGS) in PBS for 1 hour. Slides were incubated with primary antibody mouse anti-AMBN sc-271012 at dilution 1:200 in 1.5% NGS in PBS at 4°C overnight. Primary antibody was omitted in the negative control. Slides were washed three times in PBS then incubated with goat anti-mouse Alexa Fluor 488 secondary antibody 1:400 in 4% NGS in PBS for 2h at room temperature with gentle rocking. Samples were washed three times with PBS and mounted with Mowiol (Cold Spring Harbor Protocols). Rat gingiva FFPE sections were stained similarly, but using antigen retrieval overnight 60°C, primary antibody rabbit anti-AMBN DF8503 at dilution 1:200 in 2% NGS in PBS, and secondary antibody goat anti-rabbit Alexa Fluor 488 1:800 in 4% NGS in PBS.

**Confocal Imaging**

Slides were imaged using a Leica SP8 laser scanning confocal microscope. Overview images used oil immersion with HC PL APO CS2 20x/0.75 IMM objective, pixel size 0.132μm, excitation at 488nm and bandpass filter 500-550nm, with the detector gain and excitation power set to show signal from both surrounding tissue and AMBN-positive regions. Close up images used HC PL APO CS2 40x/1.30 oil objective, pixel size 0.063μm, using sequential scanning for Alexa Fluor 488 and autofluorescence. Alexa Fluor 488 was imaged with excitation 488nm, bandpass 500-550nm, and detector and laser power optimized to show only Alexa Fluor 488-positive regions. Surrounding tissue was imaged using 405nm excitation and bandpass 410-470nm to show tissue autofluorescence to aid orientation within the samples. Images were processed in ImageJ/FIJI.

**Quantitative RT-PCR**

Human normal tissue cDNA array (HMRT304, OriGene Technologies) was used to determine the expression profile of *AMBN*. The qRT-PCR was performed using a CFX96 Touch Real-Time PCR Detection System (Bio Rad). RT-PCR reactions were carried out in 10µl, and the relative mRNA level of AMBN were normalized to glyceraldehyde-3-phosphate dehydrogenase (GAPDH) and presented as ΔC_T._ The sequences of the AMBN primers used, are 5′-AGCCATGTTTCCAGGATTTG-3′ (Forward) and 5′-TGCACCTCCTTCTTCGTTCT-3′ (Reverse). IQ SYBR Green Supermix (Bio Rad; Laboratories) The GAPDH primer mix was included in the commercial tissue cDNA array and SYBR green was purchased from BioRad (IQ SYBR Green Supermix).

**
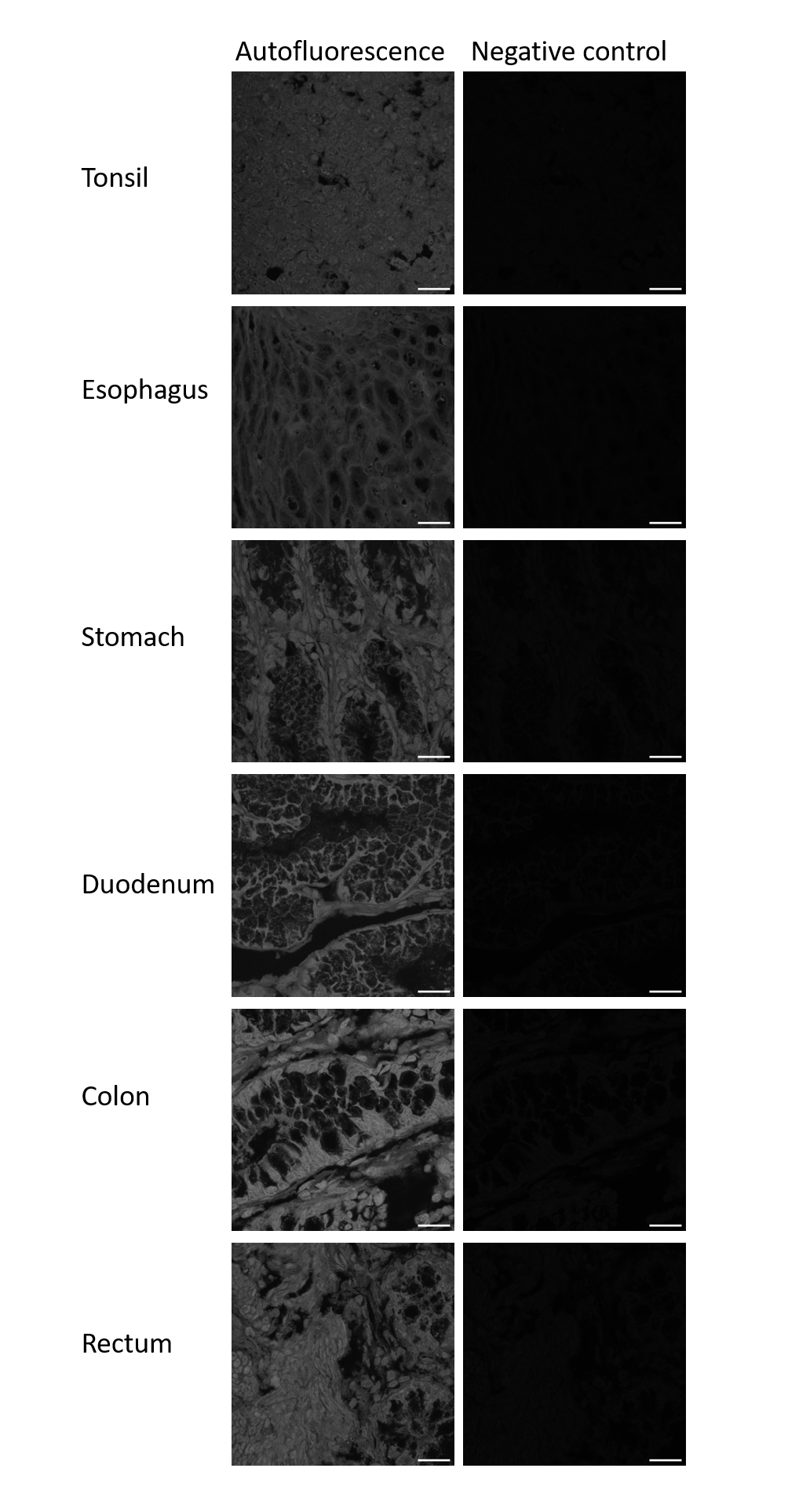
Supplementary Figure 1**

**Supplementary Figure 1 legend**

Negative control images for immunofluorescence labelling of normal human adult tissue array. Negative control samples were treated as for test samples, except that the primary antibody was omitted. Scale bar 20μm. Autofluorescence and immunolabelling images are both shown in grayscale.

**Supplementary Figure 2**

**
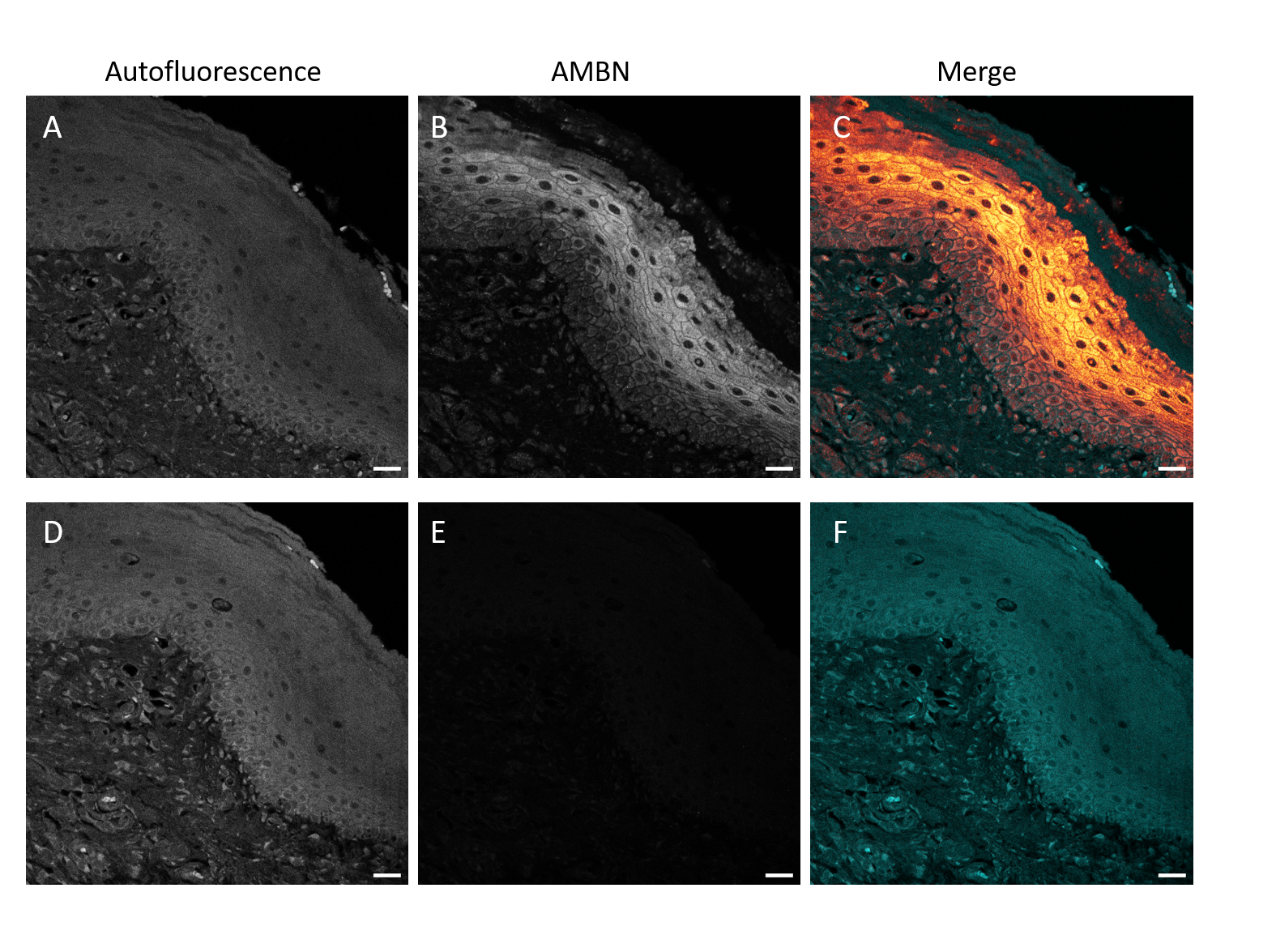
**

**Supplementary Figure 2 legend**

Rat gingiva formalin-fixed paraffin embedded sections stained by indirect autofluorescence with anti-AMBN (DF8503, 1:200, Affinity Biosciences) and goat anti-rabbit Alexa Fluor 488 (A, B, C) or negative control where primary antibody was omitted (D, E, F). Panels show tissue autofluorescence (A, D), Alexa Fluor 488 signal (B, E) or merged images (C, F). Merged images show autofluorescence in cyan and Alexa Fluor 488 signal in LUT red hot. Scale bar 20μm.
